# Profiling Antibody Responses to Infections by *Chlamydia abortus* Enables Identification of Potential Virulence Factors and Candidates for Serodiagnosis

**DOI:** 10.1371/journal.pone.0080310

**Published:** 2013-11-15

**Authors:** Vera Forsbach-Birk, Corinna Foddis, Ulrike Simnacher, Max Wilkat, David Longbottom, Gernot Walder, Christiane Benesch, Martin Ganter, Konrad Sachse, Andreas Essig

**Affiliations:** 1 Institute of Medical Microbiology and Hygiene, University Hospital, Ulm, Germany; 2 Moredun Research Institute, Edinburgh, United Kingdom; 3 Department of Hygiene and Social Medicine, Medical University of Innsbruck, Innsbruck, Austria; 4 Tierseuchenkasse Baden-Württemberg, Stuttgart, Germany; 5 Klinik für kleine Klauentiere - Stiftung Tierärztliche Hochschule, Hannover, Germany; 6 Friedrich-Loeffler-Institut (Federal Research Institute for Animal Health), Jena, Germany; Auburn University, United States of America

## Abstract

Enzootic abortion of ewes (EAE) due to infection with the obligate intracellular pathogen *Chlamydia* (C.) *abortus* is an important zoonosis leading to considerable economic loss to agriculture worldwide. The pathogen can be transmitted to humans and may lead to serious infection in pregnant women. Knowledge about epidemiology, clinical course and transmission to humans is hampered by the lack of reliable diagnostic tools. Immunoreactive proteins, which are expressed in infected animals and humans, may serve as novel candidates for diagnostic marker proteins and represent putative virulence factors. In order to broaden the spectrum of immunogenic *C. abortus* proteins we applied 2D immunoblot analysis and screening of an expression library using human and animal sera. We have identified 48 immunoreactive proteins representing potential diagnostic markers and also putative virulence factors, such as CAB080 (homologue of the “macrophage infectivity potentiator”, MIP), CAB167 (homologue of the “translocated actin recruitment protein”, TARP), CAB712 (homologue of the “chlamydial protease-like activity factor”, CPAF), CAB776 (homologue of the “Polymorphic membrane protein D”, PmpD), and the “hypothetical proteins” CAB063, CAB408 and CAB821, which are predicted to be type III secreted. We selected two putative virulence factors for further characterization, i.e. CAB080 (cMIP) and CAB063, and studied their expression profiles at transcript and protein levels. Analysis of the subcellular localization of both proteins throughout the developmental cycle revealed CAB063 being the first *C. abortus* protein shown to be translocated to the host cell nucleus.

## Introduction

*Chlamydia* (C.) *abortus* is an obligate intracellular bacterium with a biphasic developmental cycle involving infectious, spore-like elementary bodies (EBs) and metabolically active reticulate bodies (RBs), which reside and multiply within a non-fusogenic inclusion. The pathogen is the causative agent of enzootic abortion in ewes (EAE), a condition leading to considerable economic losses in sheep husbandry worldwide [1]. Transmission of the zoonotic chlamydiae to pregnant women can pose a life-threatening health risk. If not treated appropriately, the patients may develop severe septicaemia with disseminated intravascular coagulation (DIC) resulting in spontaneous abortion of the fetus [2,3]. Despite general progress in chlamydial research, our knowledge about the epidemiology of *C. abortus* infection in humans, particularly the contact persons such as shepherds, agricultural workers and the rural population, is still poor. The fact that neither individual cases nor outbreaks of abortion are usually preceded by clinical signs renders prophylaxis and prevention of the disease particularly difficult. Alongside vaccination, screening flocks and animals using serological assays could be useful to control these infections. However, only a few chlamydial antigens have been used in standardized diagnostic assays so far [4], and serological tests for detection of *C. abortus*-specific antibodies in humans are not available yet. In these circumstances, the search for *C. abortus* proteins that are expressed in the natural host and recognized by the humoral immune response appears relevant as the identified antigens broaden the spectrum of candidates for serodiagnosis and may imply identification of virulence factors, which become targets of the immune defence by interacting with host cell components. This is particularly important as tools for targeted genetic manipulation of chlamydiae are not available yet and the number of proven virulence factors of *Chlamydia* spp. is limited to a few. Virulence associated function could be assigned to the polymorphic membrane proteins (Pmps) of *C. abortus* [5], which have been identified in several pathogenic *Chlamydia* spp. and resemble autotransporters of the type V secretion system [6-8]. The members of the *pmp* gene family have been shown to be differentially expressed in the course of the developmental cycle leading to evasion of the immune response through antigenic diversity [5,6,8]. The PmpD homologue of *Chlamydia pneumoniae* has been described as an adhesion protein [9]. 

As the *C. abortus* genome is known to encode a type III secretion system (T3SS) [10], it seems reasonable to speculate that some of the chlamydial virulence factors are secreted into the host cell by use of this system. Therefore, our understanding of chlamydial pathogenesis could be improved through the identification of new T3SS effectors, the initial stage of which can be conducted *in silico* [11].

In order to identify immunoreactive *C. abortus*-specific antigens suitable for serodiagnosis, we have screened the pathogen's proteome by serological 2D immunoproteomic analysis, using sera from naturally and experimentally infected sheep, as well as from women suffering from *C. abortus* infection during pregnancy. Analysis of reactive spots was performed by mass spectrometry-based assays. The search for immunoreactive proteins was complemented by screening a *C. abortus*-specific expression library using the same ovine and human sera. 

We characterized two immunoreactive proteins, i.e. cMIP and CAB063, one of the potential T3SS effectors by gene specific expression profiling at mRNA and protein levels and demonstrated subcellular localization of the protein products by cell fractionation, indirect immunofluorescence microscopy, and transfection experiments using polyclonal antibodies generated in mice and rabbits.

## Materials and Methods

### Ethics statement

 The human and animal sera were taken from already-existing serum collections of the authors [3,12-14]. Serum samples were anonymized and their use in this study was approved by the local ethics committee of the University of Ulm (#96/09). The generation of polyclonal mice and rabbit sera against recombinant proteins was conducted in accordance with the Animal Welfare Act (TierSchG) and the institutional guidelines and was approved by the Animal Care Commission of the State government of Baden-Württemberg/Germany (#35/9185.82-2).

### Organisms and culture

Strain *C. abortus* S26/3 [10] was used throughout the study and cultured on cycloheximide-treated HeLa 229 cell monolayers (American Type Culture Collection; CCL 2.1) in Quantum 101 (PAA Laboratories, Pasching, Austria) in six-well culture plates according to standard procedures [12]. At 48 to 72 h post infection (hpi), when ≥90% of cells contained inclusions, cells were harvested and homogenized with glass beads. HeLa cell debris was removed by centrifugation at 990 x g for 10 min at 4°C. Aliquots of the supernatant were frozen at -80°C. For protein preparation aliquots were thawed and EB were purified by density gradient centrifugation as described previously [15]. EB samples were solubilized in lysis buffer (7M urea, 2M thiourea, 2% w/v CHAPS, 1% w/v DTT, and 0.8% v/v Ampholyte 3/10 from Bio-Rad, Munich, Germany) and sonicated. Protein concentrations were determined using BIO-RAD Protein Assay, (Bio-Rad) according to manufacturer’s instructions.

### Animal and human sera

Sera were taken from PCR-positive naturally infected and from experimentally infected animals at abortion [14]. In addition, two sera were taken from animals that were part of an infected flock and that were tested negative for *C. abortus* by PCR. Two human sera were obtained from pregnant women with severe *C. abortus* infections [3]. The control group comprised a serum sample of a sheep that was free of a *C. abortus* infection (“negative control”) and two samples from SPF lambs experimentally infected with *C. pecorum*, as well as two human sera collected from patients with PCR-confirmed *C. pneumoniae* or *C. trachomatis* infection, who had specific IgM and elevated IgG antibody titers, respectively (see Table 1). Sera were used after approval of the local ethical committee of the university of Ulm (#96/09).

**Table 1:** Animal and human sera.

Serum ID in Table 3	Original Serum ID	Donor	Causative Pathogen	Source / Reference
A	SG SN	**naturally** infected ewe (**abortion**)	*C. abortus*	Tierseuchenkasse Baden-Württemberg, Stuttgart, Germany
B	T167/2-4527	**naturally** infected ewe (**abortion**)	*C. abortus*	Stiftung Tierärztliche Hochschule Hannover, Germany
C	SZ178/19-7423	**naturally** infected ewe (infection is **endemic** in the flock)	*C. abortus*	Stiftung Tierärztliche Hochschule Hannover, Germany
D	SZ178/23-4207	**naturally** infected ewe (infection is **endemic** in the flock)	*C. abortus*	Stiftung Tierärztliche Hochschule Hannover, Germany
E	1291B	**experimentally** infected ewe	*C. abortus*	Livingstone et al., 2005 [14]
F	1871A	**experimentally** infected ewe	*C. abortus*	Livingstone et al., 2005 [14]
G	815B	**experimentally** infected ewe	*C. abortus*	Livingstone et al., 2005 [14]
H	968B	**experimentally** infected ewe	*C. abortus*	Livingstone et al., 2005 [14]
I	H03-02831H	**human** serum (infection during pregnancy)	*C. abortus*	Medical University of Innsbruck, Austria [3]
J	H06-12754W	**human** serum (infection during pregnancy)	*C. abortus*	Medical University of Innsbruck, Austria
K	1825A	control serum of an uninfected sheep	no infection	Moredun Research Institute, Edinburgh, UK
L	84-796	serum from a sheep **experimentally** infected with *C. pecorum* (“subtype conjunctivitis”)	*C. pecorum*	Moredun Research Institute, Edinburgh, UK
M	P787	serum from a sheep **experimentally** infected with *C. pecorum* (“subtype arthritis”)	*C. pecorum*	Moredun Research Institute, Edinburgh, UK
N	SE456	human serum from a patient with *C. pneumoniae* infection	*C. pneumoniae*	University Hospital of Ulm, Germany
O	SE20353	human serum from a patient with *C. trachomatis* infection	*C. trachomatis*	University Hospital of Ulm, Germany

### Two-dimensional immunoblot analysis and protein identification

The analysis was conducted as described previously [13] using approximately 40 µg protein of purified *C. abortus* EBs for isoelectric focusing. Sodium dodecyl sulfate-polyacrylamide gel electrophoresis (SDS-PAGE) gels were blotted onto Immobilon polyvinylidene difluoride (PVDF) membrane (Millipore, Eschborn, Germany) for immunoblot analysis or they were stained by use of the FireSilver Staining Kit (Proteome Factory, Berlin, Germany) for isolation and identification of single protein spots by mass spectrometry-based assays (see below). For immunoblot analysis human serum sample were diluted 1:200 and animal samples 1:800. Molecular weights were calculated by use of High-Range Rainbow Molecular Weight Markers” (GE Healthcare, Little Chalfont, UK).

### Protein identification

Two 2D gels were stained using the FireSilver Staining Kit (Proteome Factory) according to manufacturer’s instructions and dried. Reactive spots detected by immunoblot analysis were correlated to silver-stained proteins using Delta2D software (DECODON, Greifswald, Germany). The spots were excised from dried gels and subjected to sequence analysis. Protein identification using nanoLC-ESI-MS/MS (nano Liquid-Chromatography coupled with Electrospray–Ionization tandem mass spectrometry) was performed by Proteome Factory AG, Berlin, Germany [13,16].

### Construction and screening of an inducible genomic expression library of the strain *C. abortus* S26/3

 Genomic DNA was purified from EBs using Qiamp DNA Mini Kit (QIAGEN, Hilden, Germany), amplified by use of REPLI-g Mini Kit (QIAGEN), and partially digested with Sau3A under optimized conditions to yield fragments ranging from 0.5 to 2.0 kb. The expression vectors pTrcHisABC (Invitrogen, Darmstadt, Germany) digested with BamHI and dephosphorylated with calf alkaline phosphatase allowed cloning of the chlamydial DNA fragments under the control of the *trp-lac* promoter. Ligations were transformed into *E. coli* ER2566 and transformants were picked from Luria-Bertani (LB) agar plates containing Ampicillin (100 μg/ml). Finally, about 12,000 clones were stored in 96-well plates. The library was replicated onto nitrocellulose membranes, which were placed on LB plates containing 0.1 mM isopropyl-ß-D-thiogalactopyranoside (IPTG) to induce gene expression in cloned inserts and incubated at 30°C overnight. The colonies adhering to membranes were lysed using a solution of 10% SDS and 0.5 M sodium hydroxide. Membranes were air dried and blocked with a solution of 5% nonfat dried cow milk (GE Healthcare) overnight. Subsequent colony immunoblot analysis was conducted as described previously [12]. Reactive clones were related to the corresponding wells of microplates and streaked for single colonies again. Reactivity was confirmed by further immunoblot analysis. Finally, the chlamydial inserts of persistently reactive clones were identified by sequencing on an ABI Prism 310 automated DNA sequencer (Applied Biosystems, Darmstadt, Germany).

### Cloning, expression, and purification of CAB063, CAB080 (cMIP), and CAB668 (cTUF)

Chlamydial genomic DNA was isolated from infected HeLa cells using the Qiamp DNA Mini Kit (Qiagen). Based on the published genome sequence of *C. abortus* S26/3, the sequences of *CAB063, CAB080*, and *CAB668* were amplified by PCR using forward primers 5’-CGCGGATCCGGAATTAATCCAAGC (*CAB063*), 5’-CGCGGATCCGATCAGAGTTCTC (*CAB080*), 5’-CGCGGATCCCAACGTACTAAAC (*CAB668*), and reverse primers 5’-CCGGAATTCTTAATCCTCTGACACACTC (*CAB063*), CCGGAATTCTCATGAAGCTGTGT (*CAB080*), 5’-CCGGAATTCTGAAATCGTTCCAG (*CAB668*), all of which contained engineered BamHI and EcoRI restriction sites (underlined) at the 5’- and 3’- ends, respectively. The resulting fragments were ligated in-frame into the multi-cloning site of the protein expression vector pTrcHisA (Invitrogen) and the ligated products were used to transform *E. coli* ER2566 competent cells. Recombinant plasmid DNA was purified with QIAprep Spin Miniprep kit (Qiagen), and the constructs were checked by sequencing on an ABI Prism 310 sequencer (Applied Biosystems). The expression of the recombinant proteins was induced with 0.1 mM IPTG and incubated for 5h at 37°C. The hexahistidine-tagged proteins were affinity purified using the Protino Ni-TED 1000 kit (Macherey-Nagel, Düren, Germany), according to the manufacturer’s instructions.

### Production of antibodies

About 12µg each of the recombinant proteins CAB063, CAB080 (cMIP), and CAB668 (cTUF) were subjected to SDS-PAGE and transferred to nitrocellulose by semi-dry Western blotting. The membrane was stained with MemCode^TM^ (Thermo SCIENTIFIC, Schwerte, Germany), and the main band of each protein at 54 kDa (CAB063), 28 kDa (cMIP), and 43 kDa (cTUF), respectively, was excised, solubilized in 150 µl DMSO und used for subcutaneous immunization of mice. BALB/c mice were primed subcutaneously on day 0, boosted on days 14 and 28 with the same formulations, and bled on day 42. In addition a rabbit was immunized subcutaneously with CAB063 according to the same immunization schedule.

### Immunofluorescence microscopy

HeLa cells infected with *C. abortus* at a multiplicity of infection (MOI) of 1 were cultured on glass cover slips and fixed with either methanol or with 4% paraformaldehyde (PFA) dissolved in PBS for 10 minutes at room temperature. PFA-fixed cells were permeabilized with 1% Triton X-100 for an additional hour. After washing and blocking, the cell samples were subjected to antibody and chemical staining. Thus, 4’,6-diamidino-2-phenylindole (DAPI) stain was used for visualization of host cell nuclei and Evans blue for cytoplasmatic counterstaining. CAB063- and CAB080-specific mice sera were used as primary antibodies in 1:60 dilution and detected with Alexa Fluor 488-conjugated goat anti-mouse antibody (Invitrogen) diluted 1:400. Binding of CAB063-specific rabbit serum was detected with Alexa Fluor 488-conjugated goat anti-rabbit antibody (Invitrogen). Images were examined using a Zeiss EC "Plan-Neofluar" 63x/1,25 oil lens on an AXIO Imager M2 microscope (Carl Zeiss, Jena, Germany).

### Quantitation of gene expression using real-time polymerase chain reaction (qPCR)

HeLa cells were infected with *C. abortus* and simultaneous purification of DNA and RNA from infected monolayers was performed with the AllPrep DNA/RNA Mini Kit (Qiagen) according to the manufacturer’s instructions. Reverse transcription (RT-PCR) was performed using a Quantitect Reverse Transcription Kit (Qiagen). qPCR was conducted in a LightCycler Instrument (Roche Applied Science, Mannheim, Germany) using unique primers and probes for *CAB063*, *CAB080*, and *ompA* (*CAB048*) synthesized by TIB MOLBIOL (Berlin, Germany) (see Table 2). 

**Table 2:** LC PCR primers and probes.

Gene	Forward primer (5’ -3’)	Reverse primer (5’ -3’)	LC probe-3'-Fluorescein / 5’- LC Red640 probe
*CAB048* (*ompA*)	TGTAGGCATCACTCAAGGAATCG	CCGACTTAGTGTCAGTTGCCTG	CGTATTGGAACTCTGCTCCTAAAGTCGC / CAACCACACTCCCATAAAGCTCCG
*CAB063*	AGCATAGACTCTAATGACTCAACCG	CCCAACCTAGATGTTACTGTCTGTAA	AACGCTGGACAAATCACGAAGCACA / GAGCCAATAGCGTACAAACTTGCCTGTT
*CAB080*	AAGAAAACCTCTCCCTAGCC	CTGAAGGTTTCCCTGATATTG	AGGTGTCGTAGAAGTACAAGCTGATAAATT/ CAGTACCGTATCGTAAAAGAAGGAACGG

The amplification mixture consisted of 0.5 µM forward and reverse primer, respectively, and 0.15 µM LC and FL probes, respectively, as well as 10 µl Quantitect Probe PCR Master Mix (Qiagen) and 2 µl template cDNA, in a total volume of  20 µl. Samples were amplified using the following cycling profile: initial denaturation at 95 °C for 15 min, followed by 45 cycles of denaturation for 5 s at 95 °C, annealing for 30 s at 57°C (for CAB048 and CAB063) and at 54°C for CAB080 and elongation for 30 s at 72 °C.

Quantitation of gene expression was performed as described elsewhere [5]. Briefly, total numbers of genomes and transcripts were calculated using standard curves of the corresponding plasmid DNA (serial dilution with 10^2^ - 10^8^ copies). Transcripts were normalized against genomes for each time point. 

Based on three independent experiments, statistical error was calculated using the standard error of the mean. 

### Subcellular fractionation of HeLa cells infected with *C. abortus*

The ProteoExtractTM subcellular proteome extraction kit (Calbiochem, Darmstadt, Germany) allows the successive isolation of cytosolic proteins, organelle-specific proteins, and nuclear proteins. As previously described, chlamydial inclusions of infected HeLa cells can be detected in the “organelle fraction” [17,18]. The extraction procedure was performed according to the manufacturer’s instructions, except that for the cytoplasmic extraction of HeLa cells “Buffer I” was supplemented with 1mM NaVO3. Proteins from each of the extracted fractions were separated by SDS-PAGE in a 10% polyacrylamide gel system. Gels were electroblotted onto Immobilon polyvinylidene difluoride membrane (Millipore, Billerica, MA, USA) for immunoblotting as described previously [12]. 

### Transfection of HeLa cells

The sequences of *CAB063* and *CAB080* were amplified by PCR using the forward primers 5’- CCGGAATTCATGGGAATTAATC (*CAB063*) and 5’-CCGGAATTCATGAAAAAACAATGG (*CAB080*) as well as the reverse primers 5’- CTGGTCGACTTAATCCTCTGACAC (*CAB063*) and 5’-CTGGTCGACTCATGAAGCTGTGTTTTTG (*CAB080*) with engineered EcoRI and SalI restricton enzyme sites (underlined) at the 5’- and 3’- ends of the cloned sequence, respectively. The resulting fragment was ligated into the multi-cloning site of the pCI Mammalian Expression Vector (Promega, Mannheim, Germany). After transformation of *E. coli* ER2566 competent cells recombinant plasmid DNA of pCI-CAB063 was purified with EndoFree Plasmid Maxi Kit (Qiagen).

HeLa cells were plated on glass coverslips so that cells were approximately 60-80% confluent at the time of transfection. The transfection experiment was performed using Nanofectin (PAA) at a DNA (µg) to reagent (µl) ratio of 1:3.2 according to the manufacturer’s instructions. After 24 h cells were washed once with PBS and fixed with PFA. For CAB063 transfection additional cell samples were fixed with methanol. Coverslips were stained using CAB063- and cMIP-specific sera, respectively as described above. The experiment was performed three times yielding transfection rates of about 50%.

## Results

### Identification of 42 immunoreactive *C. abortus* proteins by 2D immunoblots

**Figure 1:**
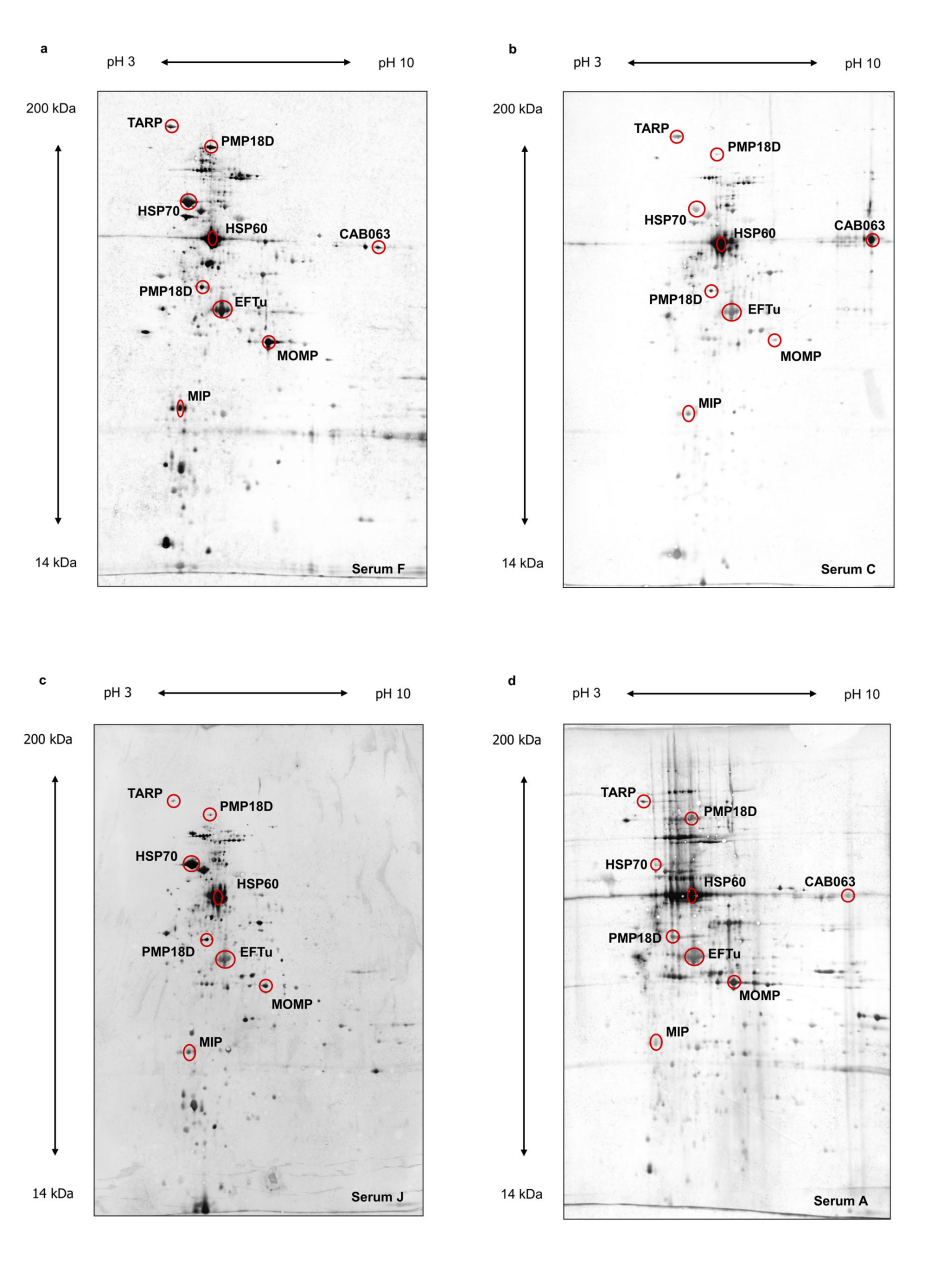
Representative examples of 2D immunoblot results. **a**, experimentally infected sheep, serum F. **b**, naturally infected sheep, infection is endemic in the flock, serum C. **c**, human sample, serum J. **d**, naturally infected sheep, abort, serum A. Serum samples refer to Table 1. Prominent spots identified as the homologues of MOMP (CAB048), HSP60 (CAB615), HSP70 (CAB237) and the elongation factor TU (CAB668) as well as the putative virulence associated proteins MIP (CAB080), PMP18D (CAB776), CAB063, and TARP (CAB167) are marked by circles.

In this study we used a proteomic approach to identify immunogenic proteins of *C. abortus*. Proteins prepared from purified elementary bodies were separated by 2D gel electrophoresis and immunoblot analysis was performed using an assortment of different sera including sheep sera from naturally and experimentally infected sheep as well as two human sera from women with severe *C. abortus* infection during pregnancy (see Figure 1). 

Immunoblot images given in Figure 1 revealed largely similar patterns showing minor differences in the total number of stained spots and individual signal intensities (see Table S1). Some prominent spots have been identified as homologues of MOMP (major outer membrane protein), the heat shock proteins HSP60 and HSP70, elongation factor Tu (EF-Tu), and PMPD (see Figure 1).

In total 42 immunoreactive protein spots could be identified by mass spectrometry based methods. Two significant protein hits were identified for each of three spots (31, 35, and 39) (see Table S1), whereas three proteins were identified following the analysis of two individual spots (CAB080 from spots 15 and 31; CAB668 from spots 1 and 39; and CAB776 from spots 9 and 26). 

Searching for *C. abortus* specific antigens, which show strong immunoblot signals with animal and human sera but no cross-reactivities using control sera, including a serum from an uninfected sheep and two sera from SPF lambs experimentally infected with *C. pecorum*, as well as two human sera from individuals with *C. pneumoniae* and *C. trachomatis* infection, respectively, revealed seven antigens: cElongation factor Ts (CAB046), cMIP (CAB080), cDNA-directed RNA polymerase subunit alpha (CAB113), cGlucose-6-phosphate isomerase (CAB703), cPMP18D (CAB776), and the two “hypothetical” proteins CAB031 and CAB821 (see Table 3). 

**Table 3:** Selection of immunoreactive *C. abortus*-proteins identified by 2D immunoblot analysis.

**Spot-no.**	**Gene symbol / Protein description**	**Locus tag**	**CalculatedMW (kDa)**	***pI***	**A**	**B**	**C**	**D**	**E**	**F**	**G**	**H**	**I**	**J**	**K**	**L**	**M**	**N**	**O**
13	CAB031 / hypothetical protein	CAB031	**87.0**	**4.54**	+++	-	+	-	+	+	++	+++	+	-	-	-	-	-	-
14	tsf / elongation factor Ts	CAB046	**30.7**	**5.42**	+	+	++	-	+++	+	(+)	++	+++	++	-	-	-	-	-
28	CAB063 / hypoth. protein	CAB063	**54.0**	**7.75**	+	+	+++	++	+++	++	+	++	-	-	-	-	-	-	-
15	mip	CAB080	**28.1**	**5.09**	(+)	+	+	-	+++	+++	++	+++	+++	++	-	-	(+)	-	-
31	mip / putative macrophage infectivity potentiator lipoprotein	CAB080	**28.1**	**5.09**	(+)	+	+	-	+	+++	+	+++	+++	++	-	-	-	-	-
2	rpoA	CAB113	**43.5**	**5.62**	+++	+++	+	+	(+)	+	(+)	+	-	+++	(+)	-	-	-	-
36	pgi	CAB703	**57.9**	**5.4**	+	-	+	+	++	+++	++	+++	+++	+++	-	-	(+)	-	-
9	pmp18D	CAB776	**163.0**	**5.27**	++	+	+	-	+++	+++	-	++	+	+	-	-	-	-	-
26	pmp18D / polymorphic outer membrane protein	CAB776	**163.0**	**5.27**	+	+++	++	-	++	+++	++	+++	+++	+++	-	-	-	-	-
37	CAB821 / hypothetical protein	CAB821	**64.0**	**5.98**	+	-	+	-	+++	+++	+++	+	(+)	+	-	-	-	-	-

For sera (A –  O) see Table 1. The signal intensities of reactive protein spots are presented semiquantitatively: (-), negative; (+), very weak, corresponding to the intensity of MIP (spot 15) in Figure 1d; + weak, corresponding to the intensity of MIP in Figure 1b; ++ moderate corresponding to the intensity of MIP in Figure 1c; +++ strong, corresponding to the intensity of MIP in Figure 1a. Column 4 shows the calculated molecular mass and column 5 the isoelectric point (*pI*) of the identified proteins. A list of all identified immunoreactive *C. abortus*-proteins is presented in Table S1.

### Identification of additional immunoreactive proteins by screening of an expression library

Six further immunogenic proteins were identified by screening of a *C. abortus* expression library using the same serum samples from infected animals and humans as previously described for 2D immunoblot analysis: CAB201 (pmp2A), CAB279 (pomp90A, alternative name: pmp12G), CAB281 (pomp91A, alternative name: pmp13G), CAB596 (pomp91B, alternative name: pmp16G), CAB575 (“putative peptide ABC transport system permease protein”), and CAB712 (homologue of the serine-type peptidase CPAF). The antigens CAB048 (MOMP), CAB080 (cMIP), CAB200 (pmp1B), and CAB750 ("putative heat shock-related exported protease") were detected by both screening of an expression library and 2D immunoblot analysis. The unique chlamydial insert of the MOMP specific clone led to high signal intensity using *C. abortus* specific animal as well as human sera. Sequence analysis revealed that this clone codes for a short protein fragment of about 9 kDa, which comprises the variable sequence “VS1”[19], the following conserved sequence, and the beginning of “VS2” (data not shown). As a total of 43 reactive clones did not contain only unique chlamydial inserts representing parts of one specific gene but in addition at least small parts of further chlamydial gene sequences, subcloning will be needed to confirm the specific immunogenicity of the encoded antigens.

### Identification of eight “unclassified” proteins by analysis of functional categories

**Figure 2:**
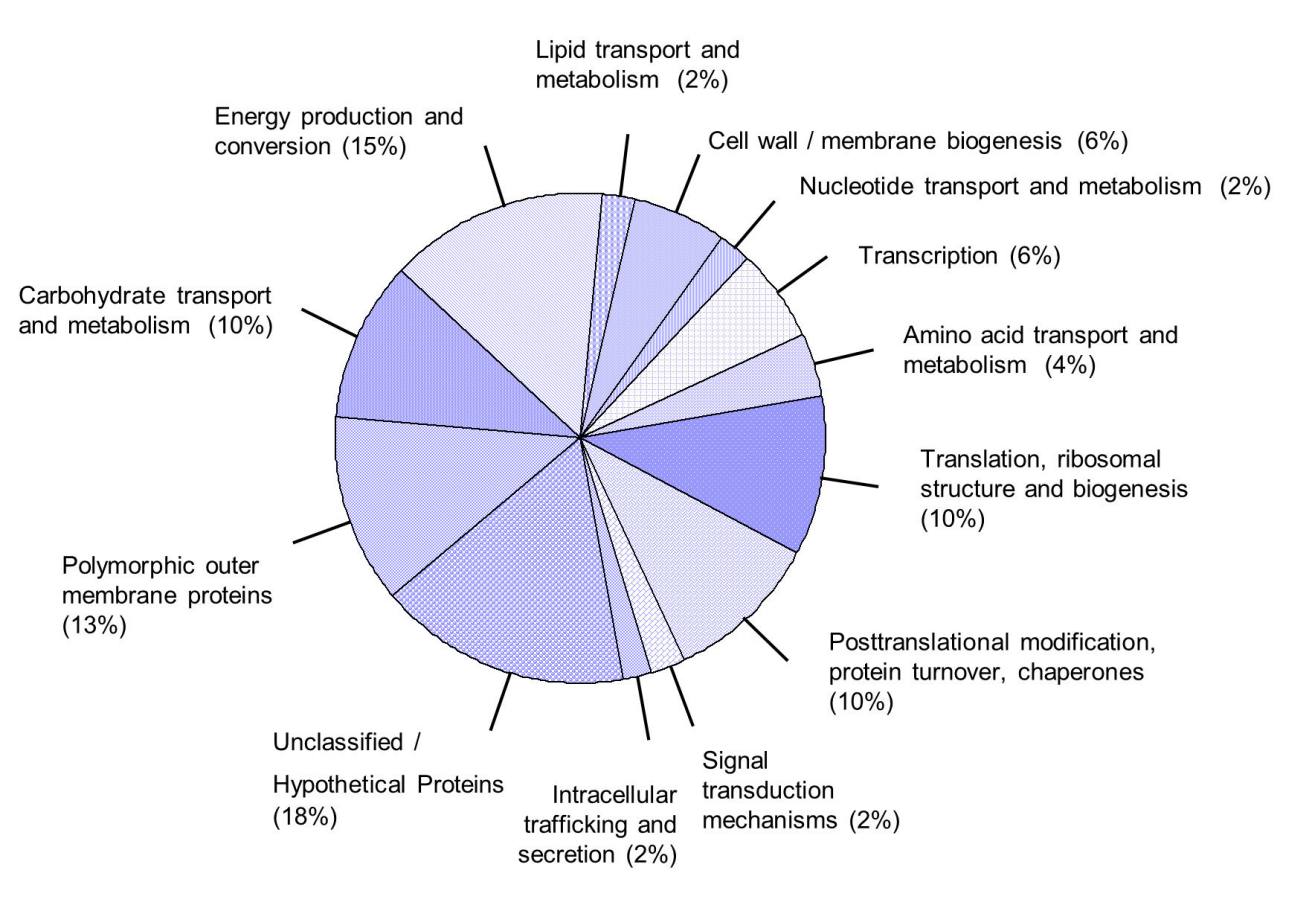
Assignment of the identified immunoreactive proteins to functional categories based on clusters of orthologous genes. For detailed information about the composition of the assigned categories, see results.

We assigned all 48 positively identified immunoreactive proteins to functional categories essentially based on clusters of orthologous genes (COGs) as listed under http://www.ncbi.nlm.nih.gov/sutils/coxik.cgi?gi=646: Carbohydrate transport and metabolism: CAB062, CAB115, CAB703, CAB841, CAB932. Energy production and conversion: CAB407, CAB654, CAB656, CAB751, CAB752, CAB816, CAB937. Lipid transport and metabolism: CAB539. Cell wall / membrane biogenesis: CAB048, CAB467, CAB712. Nucleotide transport and metabolism: CAB840. Transcription: CAB113, CAB129, CAB452. Amino acid transport and metabolism: CAB531, CAB575. Translation, ribosomal structure and biogenesis: CAB046, CAB188, CAB453, CAB668, CAB785. Posttranslational modification, protein turnover, Chaperones: CAB080, CAB237, CAB615, CAB750, CAB948. Signal transduction mechanisms: CAB031. Intracellular trafficking and secretion: CAB888. Unclassified / Hypothetical proteins: CAB015, CAB063, CAB167, CAB393, CAB395, CAB408, CAB821, CAB929. Polymorphic outer membrane proteins: CAB200, CAB201, CAB279, CAB281, CAB596, CAB776 (see Figure 2). While most of the proteins are predicted to be involved in macromolecular processes the function of eight “hypothetical proteins” (category “Unclassified / Hypothetical proteins”) remained unclear. Four of the hypothetical proteins (CAB063, CAB167, CAB408 and CAB821) are predicted to be secreted by the Type III secretion system (http://www.chlamydiaedb.org/portal/web/chlamydiaedb). CAB167 belongs to a cluster of orthologs that comprises the *C. trachomatis* specific TARP (translocated actin recruitment protein), which has been already shown to be an effector translocated by T3SS [20]. The TARP orthologs of *C. abortus* and *C. trachomatis* show 35% “Identities” and 50% “Positives” over the length of 611 amino acids that comprise three putative actin binding domains (http://blast.ncbi.nlm.nih.gov/Blast.cgi, [21]). Up to now nothing is known about the “hypothetical proteins” CAB063, CAB408 and CAB821.

### Expression profiles of *CAB063* and *CAB080* during the *C. abortus* growth cycle

We selected the “hypothetical protein” CAB063 predicted to be secreted by the Type III secretion system and CAB080 (cMIP), another putative virulence factor, which is not supposed to be secreted [22] for comparison of their expression profiles at RNA and protein levels. Both factors led to efficient antibody production after immunizing mice (CAB080, CAB063) and a rabbit (CAB063) with the corresponding recombinant protein thereby permitting analysis of the expression and the subcellular localization of the chlamydial proteins during the developmental cycle by cell fractionation, immunofluorescence microscopy, and transfection experiments.

**Figure 3:**
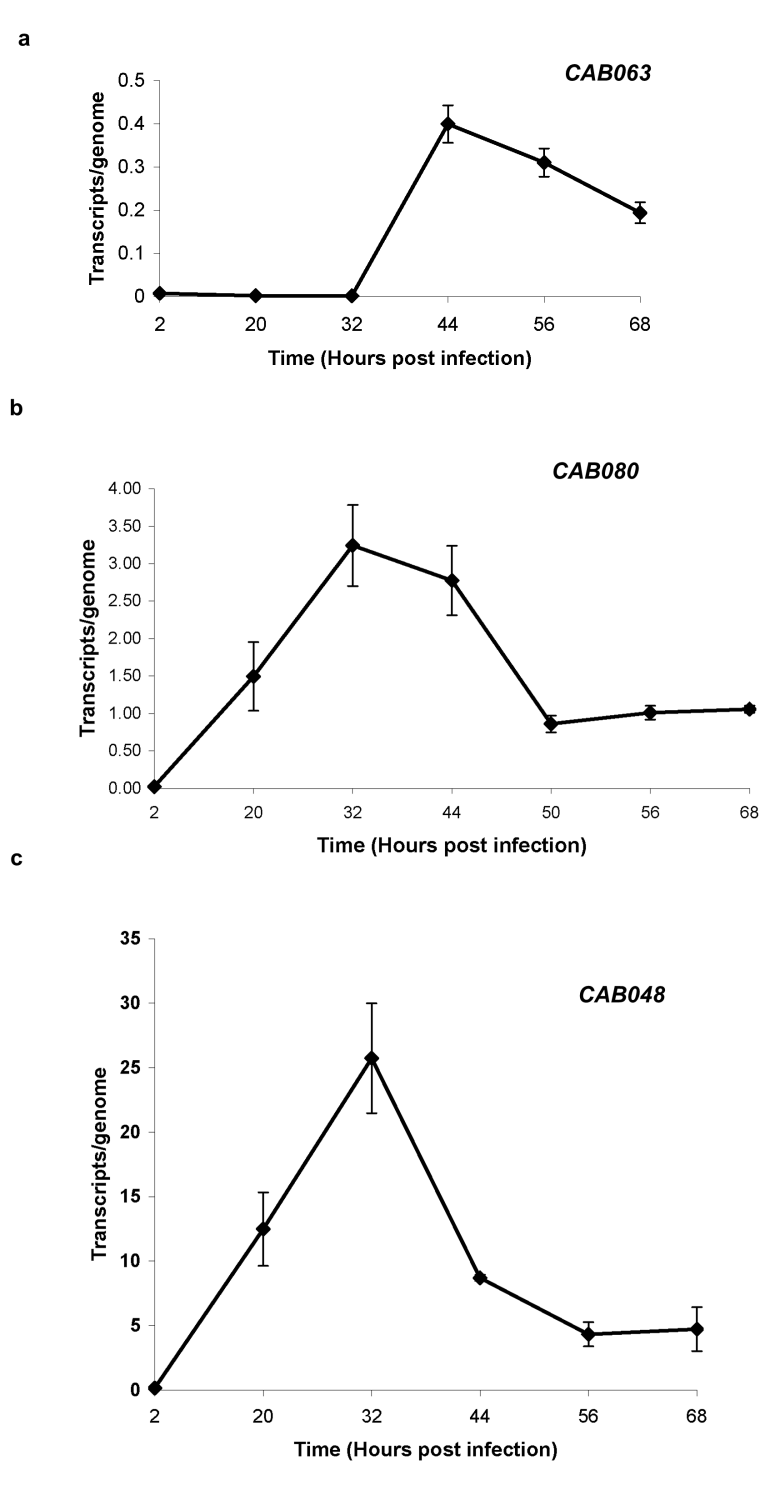
Transcriptional expression of *CAB063* (a), *CAB080* (b), and *CAB048* (**c**) **between 2 and 68 hpi**. Transcript levels were normalised relative to the genome numbers for each time point. ± SEM of three independent experiments (see M&M).

For transcriptional profiling total RNA was prepared from HeLa cells infected with *C. abortus* at 2, 20, 32, 44, 56 and 68 hpi. The number of transcripts was calculated against chlamydial genome copies for each time point as described by Wheelhouse et al. [5]. As an “internal control” we included the expression profile of *CAB048* (*ompA*), and compared it to the profile previously described [5] (Figure 3c). Both profiles turned out to be similar. Transcript expression of *ompA* showed a significant rise between 2 and 20 hpi and exhibited peak expression at 32 hpi before decreasing again. 

For *CAB063* the results of the real-time PCR assays showed no significant gene expression up to 32 hpi. Subsequently an increase led to a maximum of gene expression at 44 hpi followed by decrease of transcripts to about half of maximum at 68 hpi (Figure 3a). The expression pattern of cMIP showed maximal expression between 32 and 44 hpi (Figure 3b).

**Figure 4:**
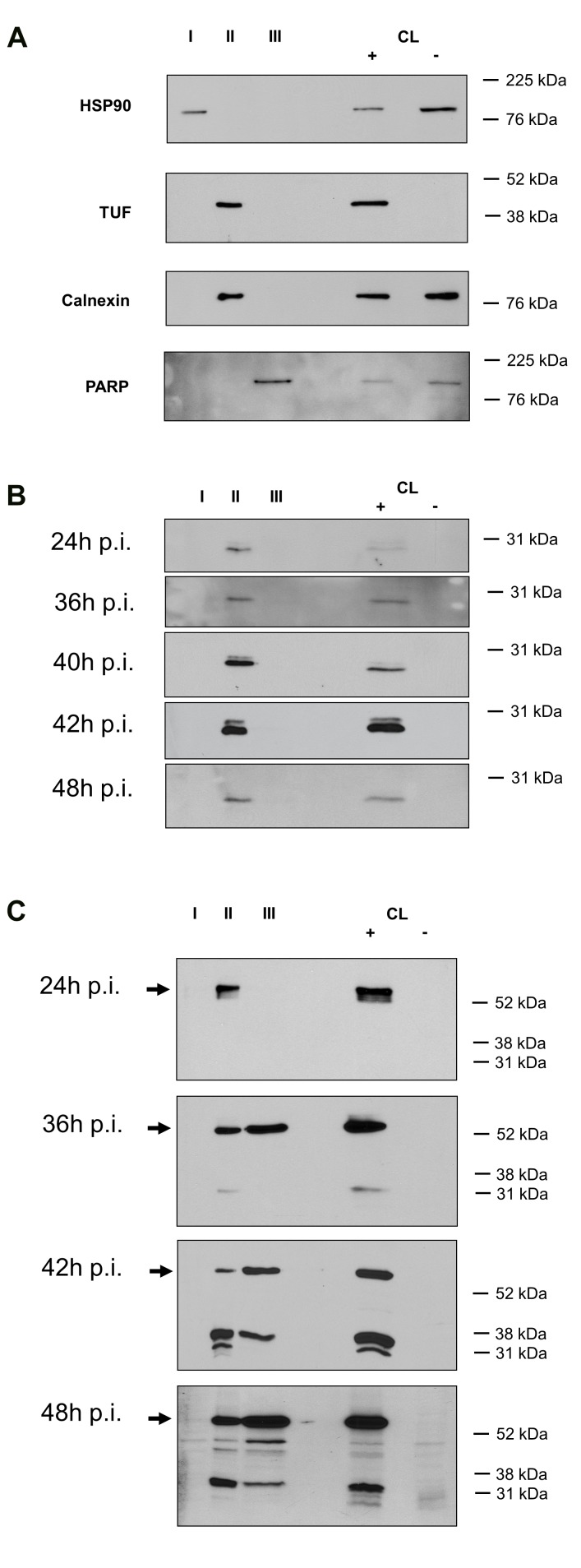
Subcellular localization of cMIP and CAB063during the developmental cycle of HeLa cells infected with *C. abortus*. I, cytosolic proteins; II, organelle specific proteins comprising chlamydial inclusions; III, nuclear proteins. CL +, lysate of HeLa cells infected with *C*. *abortus*. CL -, lysate of HeLa cells not infected with *C*. *abortus*. **a**. Selectivity of subcellular extraction was confirmed by immunoblotting with antibodies directed against the indicated marker proteins: HSP90 (cytosolic fraction, I); chlamydial elongation factor TUF and the endoplasmic reticulum protein calnexin (organelle fraction comprising chlamydial inclusions, II); nuclear Poly ADP ribose polymerase, PARP (nuclear fraction, III). **b**. Detection of CAB080 (cMIP) solely in the organelle/chlamydial inclusion fraction II of HeLa cells infected with *C*. *abortus*. **c**. Detection of CAB063 specific signals in fraction II of HeLa cells infected with *C*. *abortus* at all time points and additionally in the nuclear fraction at 36 hpi, 42 hpi, and 48 hpi. Arrows indicate the full-length protein of CAB063 (54 kDa).

In order to study the expression profile of CAB063 and cMIP at the protein level and to determine their subcellular localization during the developmental cycle *C. abortus* infected cells were harvested at 24 hpi, 36 hpi, 42 hpi, and 48 hpi. Subcellular fractionation was performed for the successive isolation of cytosolic proteins (fraction I), organelle specific proteins (fraction II), and nuclear proteins (fraction III). As already shown by others [17] we confirmed the enrichment of chlamydial inclusions of infected HeLa cells within the organelle specific fraction by use of polyclonal cTUF antibodies, which detected the chlamydial elongation factor solely in this fraction (see Figure 4a). Furthermore, selectivity of subcellular extraction could be demonstrated by immunoblotting with antibodies directed against fraction specific marker proteins such as HSP 90 (cytosolic fraction I), calnexin (organelle specific fraction II), and PARP (nuclear fraction III) (see Figure 4a). 

**Figure 5:**
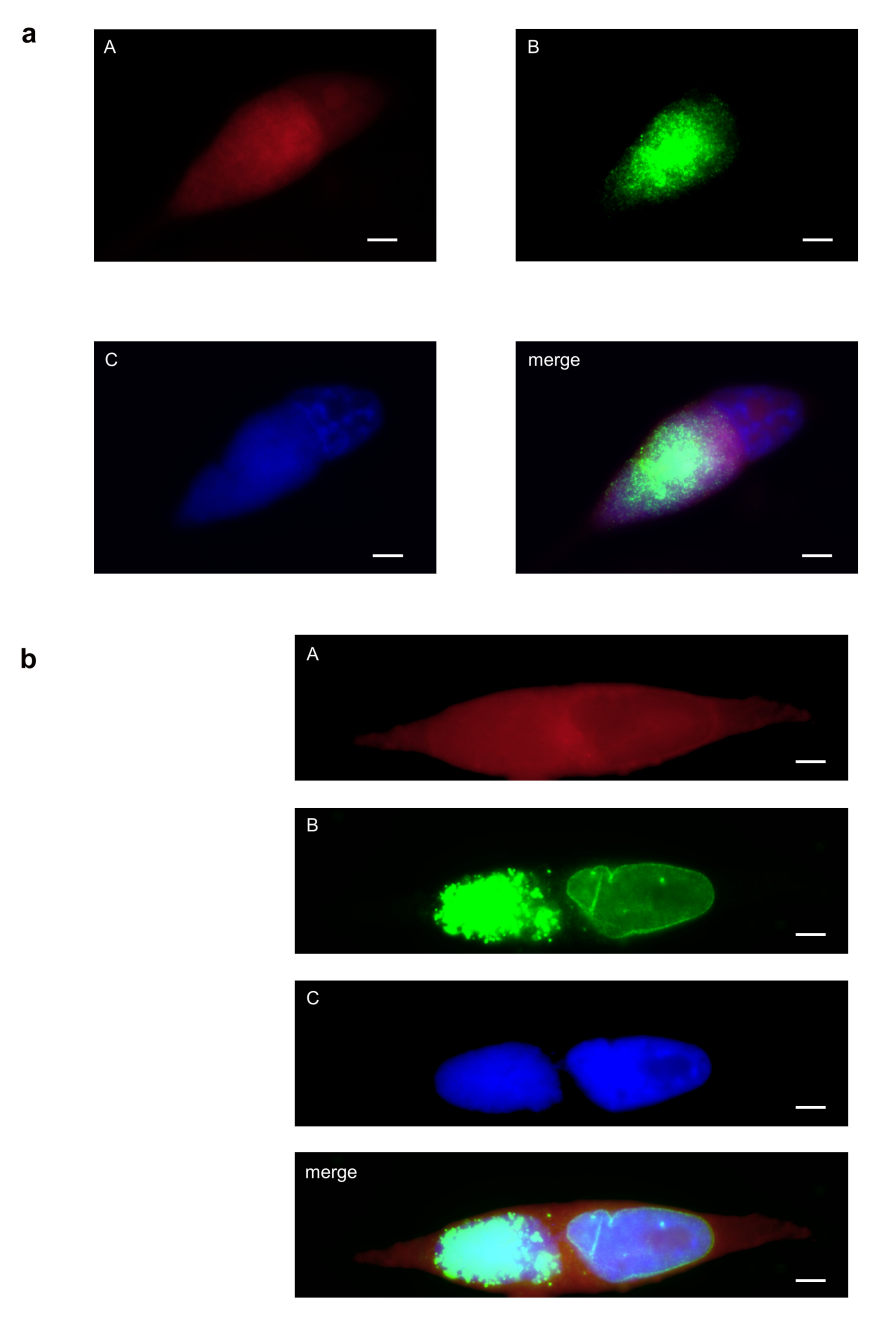
Expression and subcellular localization of cMIP and CAB063. Immunofluorescence microscopy of *C*. *abortus* infected HeLa cells fixed with PFA at 48 hpi shows localization of cMIP (Figure 5a) and CAB063 (Figure 5b) within the chlamydial inclusions and additional CAB063 specific signals in the host cell nucleus. (A) Evans blue, cytoplasmic counterstaining; (B) anti-CAB080 mouse antibodies (Figure 5a) and anti-CAB063 rabbit antibodies (Figure 5b), respectively; (C) DAPI, staining of nuclei. Scale bar = 5 µm.

**Figure 6:**
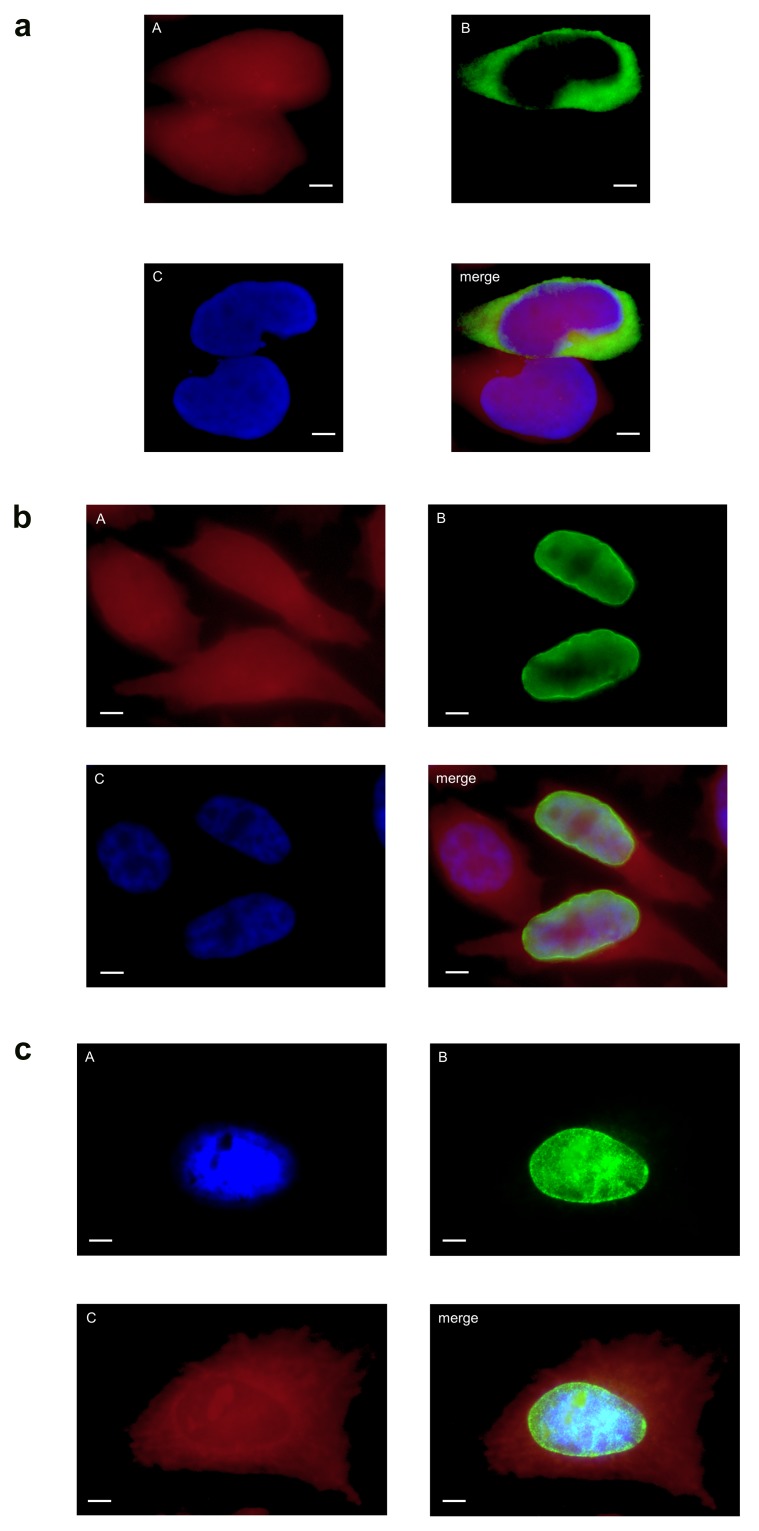
Ectopical expression of cMIP and CAB063 in HeLa cells. HeLa cells were transfected with pCI-CAB080 (a) and with pCI-CAB063 (b, c), respectively. The cells were analyzed by immunofluorescence microscopy using (A) Evans blue for cytoplasmic counterstaining, (B) anti-CAB080 mouse antibodies (a) and anti-CAB063 rabbit antibodies (b, c), respectively, and (C) DAPI to stain nuclei (c). Scale bar = 5 µm. **a**, Enrichment of cMIP (CAB080) in the cytoplasm of a transfected HeLa cell located next to a not transfected cell. **b**, **c**: Ectopically expressed CAB063 localizes to the host cell nucleus showing an intensified rim around the nucleus. b and c show representative examples of transfected cells, fixed with PFA (b, showing a not transfected and two transfected cells) and methanol (c), respectively.

At all time points analysed the full-length protein of cMIP was only found within the organelle/inclusion fraction II (see Figure 4b). This localization was confirmed by immunofluorescence microscopy of HeLa cells infected with *C. abortus* at 48 hpi (see Figure 5a). As expected the ectopic expression of cMIP in HeLa cells led to enrichment of the chlamydial protein in the cytoplasm of transfected HeLa cells (see Figure 6a). 

The full-length protein of CAB063 was detected within the organelle fraction at 24 hpi and additionally in the nuclear fraction at 36 hpi, 42 hpi, and 48 hpi (see Figure 4c). The occurrence of smaller fragments might be due to biological protein processing in chlamydiae or might just reflect some artefactual phenomenon such as insufficient inhibition of protease activities. No signal was found in the “eukaryotic cytoplasmic fraction I”. Nuclear localization of CAB063 was confirmed by immunofluorescence microscopy of *C. abortus* infected HeLa cells at 48 hpi (see Figure 5b) and by transfection experiments. As shown in Figure 6b and Figure 6c ectopic expression of CAB063 led to strong accumulation of the chlamydial protein in the nucleus of all transfected HeLa cells. 

## Discussion

To identify novel candidates for serodiagnosis as well as putative virulence factors of *C. abortus* we performed a comprehensive analysis of immunoreactive proteins in animal and human *C. abortus* infection. Combining serological proteome analysis and screening of an expression library we identified 48 immunoreactive proteins and assigned them to functional categories that comprised energy metabolism, carbohydrate metabolism, lipid metabolism and several other macromolecular processes as well as membrane proteins and “hypothetical proteins” (see Figure 2). Prominent antigens in human as well as in animal infection included MOMP, HSP60, HSP70, and elongation factor TU, most of which have been previously described as immunogenic in other chlamydial species [13,23]. In addition further immunoreactive proteins could be identified such as the homologues of CPAF (CAB712), MIP (CAB080), TARP (CAB167), and PMPD (CAB776); all of them were also discussed as virulence associated factors in other chlamydial infections [6,7,20,22,24]. Eighteen % of the identified proteins could not be assigned to a functional category. Three of these so called hypothetical proteins have been predicted to be secreted by the T3SS (CAB063, CAB408, and CAB821). This study presents the first experimental evidence of their immunogenicity and hence their expression *in vivo* in humans and animals.

 Our results show that both experimental procedures for detection of immunoreactive proteins may complement each other. 2D analysis detected full-length proteins in high resolution and led to the identification of 42 chlamydial antigens. Nevertheless, the sensitivity of this method is limited by the relative abundance of certain proteins within the analyzed EBs. Furthermore this approach can not detect proteins that are expressed only during growth within the host cell. So it was not surprising that a further six proteins were identified by screening of an expression library such as CAB712, the homologue of the *C. trachomatis* serine-type peptidase CPAF, which has been shown to be expressed only in RBs and which is described as a virulence factor that degrade host transcription factors required for major histocompatibility complex antigen expression in *C. trachomatis* [24,25]. Moreover, as most reactive library clones do not express full-length proteins but just fragments, this method also offers the chance for the identification of polypeptides comprising exclusively immunogenic domains, which subsequently may be used for further evaluation with greater numbers of sera in a diagnostic assay format. In fact, we isolated a clone coding for a 9 kDa fragment of the MOMP homologue CAB048 comprising VS1 and part of VS2 that leads to high signal intensity using *C. abortus* specific animal or human sera. Interestingly, an ELISA using the variable sequences VS1 and VS2 of MOMP has been described for diagnosis of EAE [26].

Differences in spot reactivities between naturally infected ewes with abortion, animals of flocks with endemic *C. abortus* infection, and experimentally infected ewes might be due to different time points of blood sampling after abortion, persistence of the chlamydiae in endemically-infected sheep, and/or individual differences in the immune response. In case of naturally infected sheep we even cannot exclude previous vaccination against EAE. Comparison of human and animal specific antigens revealed only few differences thereby offering the possibility to develop a combined serological testing system for human and veterinary medicine. Thus, for searching of applicable antigens we evaluated the immunoreactive proteins against the background of cross-reactivities using both human and animal control sera (see Table 1). A German study determining the prevalence of chlamydial infections in sheep flocks by PCR and DNA microarray testing identified *C. abortus* being the species most frequently found followed by *C. pecorum*, another principal chlamydial species that infect livestock [27]. Surprisingly a substantial part of mixed infections was detected. So we used not only an animal control serum from an uninfected sheep but also samples from SPF lambs experimentally infected with *C. pecorum*.

Seven chlamydial antigens (CAB031, CAB046, CAB080, CAB113, CAB703, CAB776, and CAB821) were identified that showed no or only very weak cross-reactivities with sera from humans and animals infected with other chlamydial species. If all of these proteins are suitable for specific *C. abortus* serodiagnosis has to be shown by use of larger numbers of control sera since CAB046 (Elongation factor Ts), CAB113 (DNA-directed RNA polymerase subunit alpha), and CAB703 (Glucose-6-phosphate isomerase) represent housekeeping proteins, whose homologues are widespread within prokaryotic microorganisms. However, CAB080 (Macrophage Infectivity Potentiator, cMIP), CAB776 (polymorphic outer membrane protein, pmp18D), and CAB821 (hypothetical protein, predicted to be T3SS) code for putative virulence associated proteins. Two of them, CAB080 and CAB776, have been already described as immunogenic proteins in infected ewes [28]. As shown in our study both proteins also react with human serum probes. The homologues of both proteins play an important role in pathogenicity of the human pathogen *C. trachomatis*. In *C. trachomatis* MIP has been shown to be a surface exposed lipoprotein [22] with proinflammatory activity that stimulates cytokines via Toll-like receptor 2 (TLR2)/TLR1/TLR6 and CD14 [22]. PmpD, the CAB776 homologue of *C. trachomatis*, which is also described as immunoreactive protein in humans [13] exists as a surface-associated oligomer that is suggested to enhance the chlamydial attachment and/or entry in early host cell infection. Moreover a soluble, proteolytically processed fragment of PmpD comprising a putative nuclear localization signal (NLS) may be involved in regulation of host gene expression [6,29]. 

CAB821 as well as CAB063, CAB167 and CAB408 are predicted to be secreted via T3SS (http://liferay.csb.univie.ac.at/portal/web/chlamydiaedb). CAB167 shows homology to the type III secreted effector TARP of *C. trachomatis*, which is implicated in the recruitment of actin at the site of chlamydial attachment to promote internalization into the host cell [20]. Nothing is known about the function of the “hypothetical” proteins CAB063, CAB408 and CAB821. As these potential T3SS effectors may play a role in chlamydial pathogenesis we started to analyse the expression and subcellular localization of one of these proteins, CAB063, in comparison to the MIP homologue CAB080 during the developmental cycle. 

cMIP showed similar expression patterns at RNA and protein levels revealing maximum expression between 32 and 44 hpi. By cell fractionation, the full-length protein could be only detected within the “chlamydial inclusion fraction” (fraction II) throughout the developmental cycle. This subcellular localization was confirmed by immunofluorescence microscopy of HeLa cells infected with *C. abortus*. We could not observe any enrichment of the protein in the inclusion membrane as described for the MIP homologue of *C. pneumoniae* [30]. 

Transcripts of *CAB063* were observed in the late stage of infection after 32 hpi showing only low level expression (less than 1 copy per genome). A similar low rate of expression showing less than 1 copy per genome has been already described for several polymorphic membrane proteins of *C. abortus* [5]. The expression pattern was confirmed at the protein level by subcellular fractionation of HeLa cells infected with *C. abortus* at different time points during the developmental cycle. The presence of CAB063 within the nuclear fraction corresponds to nucleus specific signals found by immunofluorescence microscopy and is consistent with the presence of a nuclear localization signal (NLS) “TRG_NLS_MonoExtN_4” (RPLKRKAP, aminoacid positions 214-221) that has been shown to be recognised by the importer protein importin-alpha [31]. Strikingly, nuclear localization of CAB063 was also observed when ectopically expressed by transfection of HeLa cells indicating that no further chlamydial proteins are necessary for translocation of CAB063 to the host cell nucleus and supporting the biological function of the identified NLS. CAB063 is the first *C. abortus* protein shown to be translocated to the host cell nucleus. To our knowledge only four chlamydial proteins with nuclear localization have been described until now [18,32,33]. While the *C. trachomatis* DUF582 proteins (CT620, CT621, CT711) are more abundant in the cytosol than in the nucleus, the “nuclear effector protein” NUE (CT737) shown to be a histone methyltransferase is localized predominantly in the nucleus. Interestingly, sequences of NUE as well as of CAB063 contain an internal nuclear localization signal. 

## Conclusion

We identified 48 immunoreactive proteins in ovine and human *C. abortus* infection including novel candidates for serodiagnosis as well as putative virulence factors. A systematic evaluation of the corresponding recombinant proteins with larger numbers of sera is on the way to confirm their diagnostic usefulness. Although the function of CAB063 remains to clarify this study presents the first experimental evidence for translocation of a *C. abortus* protein into the host cell nucleus at the end of the developmental cycle.

## Supporting Information

Table S1 **Immunoreactive *C. abortus*-proteins identified by 2D immunoblot analysis.** The signal intensities of reactive protein spots are presented semiquantitatively: (-), negative; (+), very weak, corresponding to the intensity of MIP (spot 15) in Figure 1d; +, weak, corresponding to the intensity of MIP in Figure 1b; ++, moderate corresponding to the intensity of MIP in Figure 1c; +++, strong, corresponding to the intensity of MIP in Figure 1a. Column 4 shows the calculated molecular mass and column 5 the isoelectric point (pI) of the identified proteins.
(DOC)

## References

[1] Longbottom D, Coulter LJ (2003) Animal chlamydioses and zoonotic implications. J Comp Pathol 128: 217-244. doi:10.1053/jcpa.2002.0629. PubMed: 12834606. 12834606

[2] Baud D, Regan L, Greub G (2008) Emerging role of Chlamydia and Chlamydia-like organisms in adverse pregnancy outcomes. Curr Opin Infect Dis 21: 70-76. doi:10.1097/QCO.0b013e3282f3e6a5. PubMed: 18192789. 18192789

[3] Walder G, Hotzel H, Brezinka C, Gritsch W, Tauber R et al. (2005) An unusual cause of sepsis during pregnancy: recognizing infection with chlamydophila abortus. Obstet Gynecol 106: 1215-1217. doi:10.1097/01.AOG.0000161060.69470.9c. PubMed: 16260577. 16260577

[4] Sachse K, Vretou E, Livingstone M, Borel N, Pospischil A et al. (2009) Recent developments in the laboratory diagnosis of chlamydial infections. Vet Microbiol 135: 2-21. doi:10.1016/j.vetmic.2008.09.040. PubMed: 18986778. 18986778

[5] Wheelhouse N, Aitchison K, Spalding L, Livingstone M, Longbottom D (2009) Transcriptional analysis of in vitro expression patterns of Chlamydophila abortus polymorphic outer membrane proteins during the chlamydial developmental cycle. Vet Res 40: 47. doi:10.1051/vetres/2009030. PubMed: 19454212. 19454212 PMC2704334

[6] Kiselev AO, Stamm WE, Yates JR, Lampe MF (2007) Expression, processing, and localization of PmpD of Chlamydia trachomatis serovar L2 during the chlamydial developmental cycle. PLOS ONE 2: e568. doi:10.1371/journal.pone.0000568. PubMed: 17593967. 17593967 PMC1892801

[7] Kiselev AO, Skinner MC, Lampe MF (2009) Analysis of pmpD expression and PmpD post-translational processing during the life cycle of Chlamydia trachomatis serovars A, D, and L2. PLOS ONE 4: e5191. doi:10.1371/journal.pone.0005191. PubMed: 19367336. 19367336 PMC2666266

[8] Tan C, Hsia RC, Shou H, Haggerty CL, Ness RB et al. (2009) Chlamydia trachomatis-infected patients display variable antibody profiles against the nine-member polymorphic membrane protein family. Infect Immun 77: 3218-3226. doi:10.1128/IAI.01566-08. PubMed: 19487469. 19487469 PMC2715660

[9] Wehrl W, Brinkmann V, Jungblut PR, Meyer TF, Szczepek AJ (2004) From the inside out--processing of the Chlamydial autotransporter PmpD and its role in bacterial adhesion and activation of human host cells. Mol Microbiol 51: 319-334. doi:10.1046/j.1365-2958.2003.03838.x. PubMed: 14756775. 14756775

[10] Thomson NR, Yeats C, Bell K, Holden MT, Bentley SD et al. (2005) The Chlamydophila abortus genome sequence reveals an array of variable proteins that contribute to interspecies variation. Genome Res 15: 629-640. doi:10.1101/gr.3684805. PubMed: 15837807. 15837807 PMC1088291

[11] Arnold R, Brandmaier S, Kleine F, Tischler P, Heinz E et al. (2009) Sequence-based prediction of type III secreted proteins. PLOS Pathog 5: e1000376 PubMed: 19390696. 19390696 10.1371/journal.ppat.1000376 PMC2669295

[12] Essig A, Simnacher U, Susa M, Marre R (1999) Analysis of the humoral immune response to Chlamydia pneumoniae by immunoblotting and immunoprecipitation. Clin Diagn Lab Immunol 6: 819-825. PubMed: 10548570. 10548570 10.1128/cdli.6.6.819-825.1999 PMC95782

[13] Forsbach-Birk V, Simnacher U, Pfrepper KI, Soutschek E, Kiselev AO et al. (2009) Identification and Evaluation of a Combination of Chlamydial Antigens to Support the Diagnosis of Severe and Invasive Chlamydia trachomatis Infections. Clin Microbiol Infect 16: 1237-1244. doi:10.1111/j.1469-0691.2009.03041.x. PubMed: 19723133. 19723133

[14] Livingstone M, Entrican G, Wattegedera S, Buxton D, McKendrick IJ et al. (2005) Antibody responses to recombinant protein fragments of the major outer membrane protein and polymorphic outer membrane protein POMP90 in Chlamydophila abortus-infected pregnant sheep. Clin Diagn Lab Immunol 12: 770-777. PubMed: 15939753. 15939753 10.1128/CDLI.12.6.770-777.2005 PMC1151967

[15] Caldwell HD, Kromhout J, Schachter J (1981) Purification and partial characterization of the major outer membrane protein of Chlamydia trachomatis. Infect Immun 31: 1161-1176. PubMed: 7228399. 7228399 10.1128/iai.31.3.1161-1176.1981 PMC351439

[16] Demine R, Walden P (2005) Testing the role of gp96 as peptide chaperone in antigen processing. J Biol Chem 280: 17573-17578. PubMed: 15728573. 15728573 10.1074/jbc.M501233200

[17] Hobolt-Pedersen A, Christiansen G, Birkelund S (2006) Development of a new method to discover secreted C.trachomatis proteins. In: Chernesky M Caldwell H Christiansen G Clarke IN Kaltenboeck B Eleventh International Symposium on Human Chlamydial Infections. Niagara-on-the-Lake, ON, Canada pp. 237-240.

[18] Hobolt-Pedersen AS, Christiansen G, Timmerman E, Gevaert K, Birkelund S (2009) Identification of Chlamydia trachomatis CT621, a protein delivered through the type III secretion system to the host cell cytoplasm and nucleus. FEMS Immunol Med Microbiol 57: 46-58. doi:10.1111/j.1574-695X.2009.00581.x. PubMed: 19682078. 19682078 PMC2784215

[19] Vretou E, Psarrou E, Kaisar M, Vlisidou I, Salti-Montesanto V et al. (2001) Identification of protective epitopes by sequencing of the major outer membrane protein gene of a variant strain of Chlamydia psittaci serotype 1 (Chlamydophila abortus). Infect Immun 69: 607-612. doi:10.1128/IAI.69.1.607-612.2001. PubMed: 11119563. 11119563 PMC97929

[20] Clifton DR, Fields KA, Grieshaber SS, Dooley CA, Fischer ER et al. (2004) A chlamydial type III translocated protein is tyrosine-phosphorylated at the site of entry and associated with recruitment of actin. Proc Natl Acad Sci U S A 101: 10166-10171. doi:10.1073/pnas.0402829101. PubMed: 15199184. 15199184 PMC454183

[21] Jewett TJ, Miller NJ, Dooley CA, Hackstadt T (2010) The conserved Tarp actin binding domain is important for chlamydial invasion. PLOS Pathog 6: e1000997 PubMed: 20657821. 20657821 10.1371/journal.ppat.1000997 PMC2904776

[22] Neff L, Daher S, Muzzin P, Spenato U, Gülaçar F et al. (2007) Molecular characterization and subcellular localization of macrophage infectivity potentiator, a Chlamydia trachomatis lipoprotein. J Bacteriol 189: 4739-4748. doi:10.1128/JB.01889-06. PubMed: 17449608. 17449608 PMC1913453

[23] Bunk S, Susnea I, Rupp J, Summersgill JT, Maass M et al. (2008) Immunoproteomic identification and serological responses to novel Chlamydia pneumoniae antigens that are associated with persistent C. pneumoniae infections. J Immunol 180: 5490-5498. PubMed: 18390732. 18390732 10.4049/jimmunol.180.8.5490

[24] Dong F, Sharma J, Xiao Y, Zhong Y, Zhong G (2004) Intramolecular dimerization is required for the chlamydia-secreted protease CPAF to degrade host transcriptional factors. Infect Immun 72: 3869-3875. doi:10.1128/IAI.72.7.3869-3875.2004. PubMed: 15213129. 15213129 PMC427400

[25] Zhong G, Fan P, Ji H, Dong F, Huang Y (2001) Identification of a chlamydial protease-like activity factor responsible for the degradation of host transcription factors. J Exp Med 193: 935-942. doi:10.1084/jem.193.8.935. PubMed: 11304554. 11304554 PMC2193410

[26] Salti-Montesanto V, Tsoli E, Papavassiliou P, Psarrou E, Markey BK et al. (1997) Diagnosis of ovine enzootic abortion, using a competitive ELISA based on monoclonal antibodies against variable segments 1 and 2 of the major outer membrane protein of Chlamydia psittaci serotype 1. Am J Vet Res 58: 228-235. PubMed: 9055966. 9055966

[27] Lenzko H, Moog U, Henning K, Lederbach R, Diller R et al. (2011) High frequency of chlamydial co-infections in clinically healthy sheep flocks. BMC. Vet Res 7: 29. 10.1186/1746-6148-7-29 PMC3125319 21679409

[28] Marques PX, Souda P, O'Donovan J, Gutierrez J, Gutierrez EJ et al. (2010) Identification of immunologically relevant proteins of Chlamydophila abortus using sera from experimentally infected pregnant ewes. Clin Vaccine Immunol 17: 1274-1281. doi:10.1128/CVI.00163-10. PubMed: 20554807. 20554807 PMC2916250

[29] Swanson KA, Taylor LD, Frank SD, Sturdevant GL, Fischer ER et al. (2009) Chlamydia trachomatis polymorphic membrane protein D is an oligomeric autotransporter with a higher-order structure. Infect Immun 77: 508-516. doi:10.1128/IAI.01173-08. PubMed: 19001072. 19001072 PMC2612253

[30] Herrmann M, Schuhmacher A, Mühldorfer I, Melchers K, Prothmann C et al. (2006) Identification and characterization of secreted effector proteins of Chlamydophila pneumoniae TW183. Res Microbiol 157: 513-524. doi:10.1016/j.resmic.2005.12.005. PubMed: 16797933. 16797933

[31] Dinkel H, Michael S, Weatheritt RJ, Davey NE, Van Roey K et al. (2012) ELM--the database of eukaryotic linear motifs. Nucleic Acids Res 40: D242-D251. doi:10.1093/nar/gks559. PubMed: 22110040. 22110040 PMC3245074

[32] Muschiol S, Boncompain G, Vromman F, Dehoux P, Normark S et al. (2011) Identification of a family of effectors secreted by the type III secretion system that are conserved in pathogenic Chlamydiae. Infect Immun 79: 571-580. doi:10.1128/IAI.00825-10. PubMed: 21078856. 21078856 PMC3028825

[33] Pennini ME, Perrinet S, Dautry-Varsat A, Subtil A (2010) Histone methylation by NUE, a novel nuclear effector of the intracellular pathogen Chlamydia trachomatis. PLOS Pathog 6: e1000995 PubMed: 20657819. 20657819 10.1371/journal.ppat.1000995 PMC2904774

