# Supplementary material for: Profiling Antibody Responses to Infections by Chlamydia abortus Enables Identification of Potential Virulence Factors and Candidates for Serodiagnosis
Source: PLoS One. 2013 Nov 15;8(11):e80310. doi: 10.1371/journal.pone.0080310 (PMC3829881; doi:10.1371/journal.pone.0080310)
Supplement: Table S1 — Immunoreactive C. abortus-proteins identified by 2D immunoblot analysis. The signal intensities of reactive protein spots are presented semiquantitatively: (-), negative; (+), very weak, corresponding to the intensity of MIP (spot 15) in Figure 1d; +, weak, corresponding to the intensity of MIP in Figure 1b; ++, moderate corresponding to the intensity of MIP in Figure 1c; +++, strong, corresponding to the intensity of MIP in Figure 1a. Column 4 shows the calculated molecular mass and column 5 the isoelectric point (pI) of the identified proteins. (DOC) [file pone.0080310.s001.doc]

| **Spot-no.** | **Gene symbol / Protein description** | **Locus tag** | **Calculated**  **MW (kDa)** | ***pI*** | **A** | **B** | **C** | **D** | **E** | **F** | **G** | **H** | **I** | **J** | **K** | **L** | **M** | **N** | **O** |
| --- | --- | --- | --- | --- | --- | --- | --- | --- | --- | --- | --- | --- | --- | --- | --- | --- | --- | --- | --- |
| 35 | CAB015 / hypoth. protein | CAB015 | **67.3** | **4.98** | + | - | ++ | ++ | +++ | +++ | +++ | +++ | +++ | - | + | (+) | (+) | - | - |
| 13 | CAB031 / hypothetical protein | CAB031 | **87.0** | **4.54** | +++ | - | + | - | + | + | ++ | +++ | + | - | - | - | - | - | - |
| 14 | tsf / elongation factor Ts | CAB046 | **30.7** | **5.42** | + | + | ++ | - | +++ | + | (+) | ++ | +++ | ++ | - | - | - | - | - |
| 10 | ompA / major outer membrane protein precursor (Momp) | CAB048 | **37.8** | **8.1** | +++ | (+) | (+) | (+) | (+) | +++ | ++ | +++ | +++ | +++ | (+) | - | - | - | ++ |
| 40 | pgk / phosphoglycerate kinase | CAB062 | **43.2** | **5.93** | - | (+) | ++ | + | - | +++ | - | - | ++ | + | - | - | - | - | - |
| 28 | CAB063 / hypoth. protein | CAB063 | **54.0** | **7.75** | + | + | +++ | ++ | +++ | ++ | + | ++ | - | - | - | - | - | - | - |
| 15 | mip / putative macrophage infectivity potentiator lipoprotein | CAB080 | **28.1** | **5.09** | (+) | + | + | - | +++ | +++ | ++ | +++ | +++ | ++ | - | - | (+) | - | - |
| 31 | mip / putative macrophage infectivity potentiator lipoprotein | CAB080 | **28.1** | **5.09** | (+) | + | + | - | + | +++ | + | +++ | +++ | ++ | - | - | - | - | - |
| 2 | rpoA / DNA-dir. RNA polymerase subunit alpha | CAB113 | **43.5** | **5.62** | +++ | +++ | + | + | (+) | + | (+) | + | - | +++ | (+) | - | - | - | - |
| 30 | gapA / glyceraldehyde-3-phosphate dehydrogenase | CAB115 | **36.3** | **6.12** | + | + | - | + | - | + | (+) | - | +++ | ++ | - | - | - | - | - |
| 32 | rho / transcription termination factor Rho | CAB129 | **51.7** | **7.15** | + | + | ++ | + | - | - | - | - | - | - | - | - | - | - | - |
| 12 | CAB167 / hypoth. Protein (TARP-homolog) | CAB167 | **91.3** | **4.77** | ++ | ++ | ++ | ++ | +++ | +++ | + | ++ | ++ | + | + | - | (+) | - | (+) |
| 7 | CAB188 / elongation factor G (cEF G) | CAB188 | **76.8** | **5.22** | - | + | +++ | +++ | - | + | + | +++ | +++ | +++ | - | ++ | (+) | - | - |
| 8 | pmp1B / polymorphic outer membrane protein | CAB200 | **189.9** | **6.26** | +++ | + | +++ | ++ | +++ | +++ | ++ | +++ | +++ | +++ | - | + | - | (+) | - |
| 6 | dnaK / molecular chaperone DnaK (cHSP 70) | CAB237 | **71.0** | **4.98** | (+) | + | + | ++ | +++ | +++ | ++ | +++ | +++ | +++ | + | ++ | (+) | - | ++ |
| 17 | CAB393 / hypoth. protein | CAB393 | **30.4** | **5.06** | - | - | (+) | - | (+) | ++ | - | ++ | ++ | (+) | - | - | - | - | - |
| 22 | CAB395 / hypoth.l protein | CAB395 | **18.3** | **5.04** | (+) | +++ | +++ | + | + | ++ | +++ | +++ | ++ | +++ | - | + | - | + | (+) |
| 39 | sucB / dihydrolipoamide succinyltransferase | CAB407 | **40.3** | **5.17** | + | + | + | - | - | ++ | - | - | +++ | + | - | - | - | - | + |
| 33 | CAB408 / hypoth. protein | CAB408 | **27.9** | **6.59** | + | ++ | - | - | + | ++ | - | ++ | + | (+) | (+) | - | - | - | - |

| 25 | nusA / transcription elongation factor NusA | CAB452 | **48.7** | **5.07** | ++ | + | +++ | ++ | (+) | ++ | + | +++ | +++ | ++ | - | + | + | - | - |
| --- | --- | --- | --- | --- | --- | --- | --- | --- | --- | --- | --- | --- | --- | --- | --- | --- | --- | --- | --- |
| 5 | rpsA / 30s ribosomal protein S1 | CAB453 | **64.9** | **5.08** | ++ | + | + | (+) | (+) | +++ | (+) | (+) | ++ | +++ | (+) | - | + | - | ++ |
| 21 | CAB467 / hypoth. protein | CAB467 | **20.3** | **4.9** | +++ | (+) | ++ | - | ++ | +++ | +++ | +++ | ++ | - | - | (+) | - | (+) | - |
| 42 | CAB531 / ABC transporter, ATP-binding component | CAB531 | **25.4** | **6.25** | + | + | - | - | - | ++ | - | - | + | ++ | - | - | - | - | - |
| 20 | CAB539 / acetyl-CoA carboxylase biotin carboxyl carrier protein subunit | CAB539 | **18.2** | **4.93** | (+) | (+) | - | - | + | ++ | - | - | +++ | - | - | - | - | - | - |
| 3 | groEL / chaperonin GroEL (cHSP60) | CAB615 | **53.1** | **5.12** | +++ | +++ | +++ | ++ | +++ | +++ | +++ | +++ | +++ | +++ | ++ | +++ | +++ | ++ | ++ |
| 35 | atpA / V-type ATP synthase subunit A | CAB654 | **65.7** | **5.04** | + | - | ++ | ++ | +++ | +++ | +++ | +++ | +++ | - | + | (+) | (+) | - | - |
| 19 | CAB656 / V-type ATP synthase subunit E | CAB656 | **22.8** | **5.19** | - | (+) | - | - | (+) | - | - | - | (+) | + | - | - | - | - | ++ |
| 1 | tuf / elongation factor Tu | CAB668 | **43.2** | **5.35** | ++ | ++ | ++ | + | +++ | +++ | (+) | ++ | ++ | ++ | (+) | - | (+) | (+) | ++ |
| 39 | tuf / elongation factor Tu | CAB668 | **43.2** | **5.36** | + | + | + | - | - | ++ | - | - | +++ | + | - | - | - | - | + |
| 36 | pgi / glucose-6-phosphate isomerase | CAB703 | **57.9** | **5.4** | + | - | + | + | ++ | +++ | ++ | +++ | +++ | +++ | - | - | (+) | - | - |
| 24 | CAB750 / putative heat shock-related exported protease | CAB750 | **52.0** | **6.43** | +++ | ++ | +++ | - | +++ | +++ | - | - | +++ | +++ | - | (+) | - | - | ++ |
| 11 | sucD / succinyl-CoA synthetase subunit alpha | CAB751 | **30.6** | **5.77** | +++ | + | ++ | - | + | + | - | - | - | +++ | - | (+) | - | - | + |
| 38 | sucC / succinyl-CoA synthetase subunit beta | CAB752 | **41.8** | **5.04** | - | - | - | + | - | ++ | - | - | +++ | + | - | - | - | - | + |
| 9 | pmp18D / polymorphic outer membrane protein | CAB776 | **163.0** | **5.27** | ++ | + | (+) | - | +++ | +++ | - | ++ | + | + | - | - | - | - | - |
| 26 | pmp18D / polymorphic outer membrane protein | CAB776 | **163.0** | **5.27** | + | +++ | ++ | - | ++ | +++ | ++ | +++ | +++ | +++ | - | - | - | - | - |
| 41 | rplI / 50S ribosomal protein L9 | CAB785 | **18.9** | **7.88** | + | - | - | + | ++ | + | (+) | - | - | - | - | - | - | - | - |
| 16 | CAB816 / inorganic pyrophosphatase | CAB816 | **24.3** | **5.05** | + | + | - | - | (+) | - | - | + | - | - | (+) | - | - | - | - |
| 37 | CAB821 / hypoth. protein | CAB821 | **64.0** | **5.98** | + | - | + | - | +++ | +++ | +++ | + | (+) | + | - | - | - | - | - |
| 29 | CAB840 / AMP nucleosidase | CAB840 | **32.4** | **7.15** | + | (+) | - | (+) | - | + | (+) | + | + | ++ | (+) | - | - | - | - |
| 4 | CAB841 / transketolase | CAB841 | **73.4** | **5.62** | + | (+) | - | - | - | ++ | - | - | - | (+) | (+) | - | (+) | - | - |
| 18 | clpP / ATP-dependent Clp protease proteolytic subunit | CAB888 | **22.3** | **5.24** | (+) | + | (+) | - | - | - | - | - | (+) | + | - | - | (+) | - | - |
| 31 | CAB 929 / hypoth. protein | CAB929 | **21.2** | **5.28** | (+) | + | + | - | + | +++ | + | +++ | +++ | ++ | - | - | - | - | - |
| 27 | eno / phosphopyruvate hydratase | CAB932 | **45.4** | **4.6** | + | ++ | (+) | ++ | (+) | +++ | + | + | + | +++ | ++ | - | (+) | - | - |
| 23 | sdhA / succinate dehydrogenase flavoprotein subunit | CAB937 | **69.7** | **7.23** | +++ | + | + | + | - | ++ | - | - | + | + | - | - | - | - | - |
| 34 | CAB948 / putative alkyl hydro-peroxide reductase | CAB948 | **22.0** | **4.92** | - | - | - | - | ++ | +++ | - | +++ | (+) | +++ | + | - | - | - | - |
